# Alternative Polyadenylation Is a Novel Strategy for the Regulation of Gene Expression in Response to Stresses in Plants

**DOI:** 10.3390/ijms24054727

**Published:** 2023-03-01

**Authors:** Jing Wu, Ligeng Ma, Ying Cao

**Affiliations:** College of Life Sciences, Capital Normal University, Beijing 100048, China

**Keywords:** gene expression, alternative polyadenylation, stress response, plants

## Abstract

Precursor message RNA requires processing to generate mature RNA. Cleavage and polyadenylation at the 3′-end in the maturation of mRNA is one of key processing steps in eukaryotes. The polyadenylation (poly(A)) tail of mRNA is an essential feature that is required to mediate its nuclear export, stability, translation efficiency, and subcellular localization. Most genes have at least two mRNA isoforms via alternative splicing (AS) or alternative polyadenylation (APA), which increases the diversity of transcriptome and proteome. However, most previous studies have focused on the role of alternative splicing on the regulation of gene expression. In this review, we summarize the recent advances concerning APA in the regulation of gene expression and in response to stresses in plants. We also discuss the mechanisms for the regulation of APA for plants in the adaptation to stress responses, and suggest that APA is a novel strategy for the adaptation to environmental changes and response to stresses in plants.

## 1. Introduction

An essential step in the maturation of mRNA is 3′-end-processing. The 3′-end carries a series of adenine residues called the polyadenylation (poly(A)) tail in almost all eukaryotic mRNAs. The maturation of a RNA from a protein-coding gene requires the addition of the poly(A) tail at its 3′-end, and this process is highly conserved in eukaryotes [1,2]. As a level of post-transcriptional regulation of gene expression, 3′-end-processing affects many aspects of the regulation of gene expression, including nuclear export, stability, translation efficiency, and protein localization of an mRNA [3,4,5,6].

**Figure 1:**
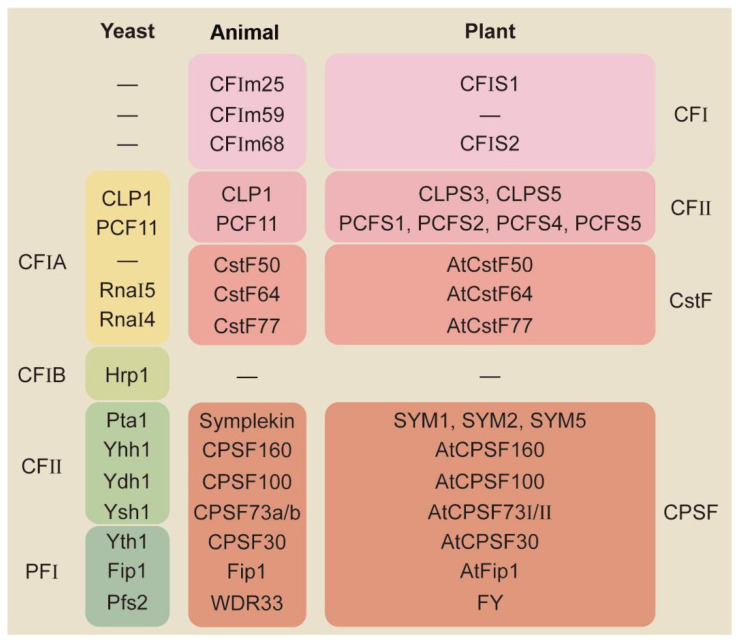
Core factors involved in 3′-end-processing. The core cleavage and polyadenylation factors are similar and can be divided into four sub-complexes in yeast, animals, and plants.

The 3′-end-processing of an mRNA in eukaryotes can be divided into two steps, cleavage and polyadenylation, which are accomplished by a large 3′-end-processing complex that includes four sub-complexes (Figure 1), and is very conserved in yeast, animals, and plants [7,8]. The regulation of polyadenylation involves a complicated interplay between the numerous cis-elements surrounding the poly(A) site and trans-acting factors, of which several are RNA-binding proteins. In vertebrates, the most prominent polyadenylation signal is the A(A/U)UAAA motif, typically located 15–30 nt upstream of the poly(A) site, and upwards of 80% of genes have this consensus sequence feature at the 3′-end of pre-mRNA which can be recognized by the CPSF (cleavage and polyadenylation specificity factor) complex [9]; at the same time, the CstF (cleavage stimulation factor) complex binds to the U-rich and GU-rich motifs downstream of the poly(A) site [10], and the CFI (cleavage factor I) complex binds to the UGUA motif upstream of the poly(A) site [11]. Compared to the three sub-complexes, the ability of the CFII (cleavage factor II) complex to bind RNA has been less studied.

The structure of the cleavage and polyadenylation complexes from yeast and animals shares roughly similar core components, yet with some differences (Figure 1), with yeast lacking the CFI complex and Cst50 that is present in vertebrates, while vertebrates lack the Hrp1 that is in yeast [12,13,14,15,16,17,18,19]. In contrast, our understanding in higher plants relies mainly on the analysis of their homologous proteins in yeast and animals [20,21,22,23,24,25,26,27,28,29,30]. Based on the present understanding, it seems that the overall composition of the 3′-end-processing complex from higher plants is relatively similar to that of vertebrates; it misses the CFIm59 in the CFI complex [20]. Another striking feature is the large number of gene duplications for genes encoding homologous proteins for the 3′-end-processing complex, especially for the homologous proteins of PCF11 and PAP1, with four each (Figure 1), which also implies that the regulatory mechanisms of polyadenylation in plants may be more exquisite and complicated [22,31].

In eukaryotes, most genes have more than one poly(A) site, and the presence of two or more poly(A) sites in a gene leads to the production of different isoforms of transcripts, which is known as alternative polyadenylation (APA) [32]. APA is present in more than 84% of yeast genes and more than 70% of animal genes [33,34,35], while it is present more than 70% of genes in the lower plant *Chlamydomonas reinhardtii* and the model plant *Arabidopsis thaliana*, and at least 50% of rice exhibits APA [36,37,38]. This suggests that APA is widespread in eukaryotes and implies that this mode of regulation is important for the regulation of gene expression. When APA occurs in upstream regions of the mRNA, it often leads to the generation of a truncated protein, thereby affecting the function of a full-length protein; however, when it occurs in 3′UTR, it may lead to a change in the stability of mRNA or translation efficiency [39].

Most previous studies have focused on the transcriptional and splicing regulation of gene expression [40,41]. In recent years, 3′-end-processing has also attracted much more attention. In particular, the advance in the technology of 3′-end-specific sequencing has given us the opportunity to gain insight into the mechanisms of 3′-end formation. Here, we discuss the molecular mechanism of APA-regulating gene expression under stress, and summarize the recent advance in the role of APA in response to stresses in plants.

## 2. Molecular Mechanisms for APA-Mediated Responses

The mechanisms for the APA-mediated stress response have rarely been reported in plants; however, there are some reports from animals and yeast for the APA-mediated regulation of gene function. We think the overall molecular mechanism is similar for APA-mediated gene function among plants, animals, and yeast, as most of the components of the APA complex are homologs among animals, yeast, and plants. Therefore, a discussion of the possible molecular mechanisms in the context of animal and yeast studies is provided.

### 2.1. Influence Full-Length Transcripts

The poly(A) tail is added to the newly generated mRNA to indicate the termination of transcription and therefore determines the coding region of a protein. There are fewer studies on 5′UTR APA and CDS APA; more extensively studied is intronic APA, where polyadenylation occurring at the intron generally quickly encounters the termination codon to form a truncated protein, such that the truncated protein may be partially active or completely lose function [42]. Intronic APA events are common in human immune cells and severely dysregulated in cancer cells [43,44], suggesting an important role of intronic APA in the precise regulation of gene expression.

Intronic APA events are often associated with competition between splicing and 3′-end-processing machinery on introns, even though eukaryote gene expression is generally considered to be co-transcribed [45]. There are interactions between splicing factor and polyadenylation factor [46,47], which may underlie the competition between them. Intronic APA events often occur in the first two introns, which often produce abnormally transcript-encoding short truncated protein and thus reduce the proportion of functional transcripts [48], or play a dominant negative role as the truncated protein inhibits the function of the full-length protein [49].

A certain percentage of introns are not fully spliced and a cryptic polyadenylation signal is recognized in the retained introns, which produces the truncated proteins under stress. Those retained introns are characterized by a length greater than the average length of introns, which may be close to the 5′UTR, implying that introns with weaker splicing signals may undergo intron retention under stress and be recognized by the 3′-end-formation machinery, which may act as competition for the function of full length and truncated proteins [50,51,52].

### 2.2. Influence RNA Fate and Translation Efficiency

In contrast to intronic APA, APA occurring on the 3′UTR does not change the protein-coding sequence, but this does not mean that 3′UTR APA produces weaker effects than other forms of APA for the function of the target gene [53]. On the contrary, 3′UTR APA can alter key sequences that regulate RNA export and translation efficiency, and therefore, the regulation of 3′UTR length by APA is an important determinant for RNA fate determination. This mode of regulation is general, as more than 50% of the genes in the human genome show 3′UTR APA events [35].

#### 2.2.1. RNA Stability

Studies in yeast have shown that mRNA stability is negatively correlated to the length of 3′UTR, with transcripts with short half-lives being twice as long as the most stable transcripts [54]. The 3′UTR contains multiple cis-elements and is subject to complex regulation by trans-acting factors, such as microRNA or RNA-binding protein [55].

The more studied factors are the role of microRNA on the stability of mRNA with 3′UTR APA. MicroRNAs are short RNAs of about 20 nucleotides in size that regulate the post-transcriptional silencing of target genes [56]. The 3′UTR APA event often alters the sequence of the UTR in the mRNA to contain or not contain the microRNA-binding site. For example, Hsp70.3 promotes the use of the proximal site in the 3′UTR, which likewise increases the expression of Hsp70.3, thereby improving cell survival under heat shock conditions [57]. The shortening in *NLRP3* 3′UTR leads to the overactivation of *NLRP3* and exacerbates the inflammatory response [58]. The up-regulation of the gene expression level for the genes with 3′UTR shortening is caused by avoiding microRNA-mediated degradation when shortening the 3′UTR.

In addition to the microRNA, RNA-binding protein also acts via the 3′UTR as a post-transcriptional regulator. The binding of RNA-binding protein to mRNA may lead to the degradation of mRNA. The 3′UTR contains multiple cis-elements, ARE (Adenylate/uridylate Reich element) is the most well-known of these, being present in 5–8% of human genes and involved in regulating many important physiological processes [59]. Mouse cells use the proximal poly(A) site under arsenic stress and enhance the degradation of long 3′UTR transcription during recovery. The degradation of long 3′UTR transcription is due to the binding of TIA1 to the U-rich motif of the long 3′UTR, promoting SG recruitment and leading to enhanced mRNA decay [60]. Thus, the use of the proximal poly(A) site under stress facilitates the retention of transcript abundance.

#### 2.2.2. RNA Export

The transport of mRNA from the nucleus to the cytoplasm is a key process in the expression of genes in eukaryotes, and this process is closely controlled by the 3′UTR [61]. Biotic and abiotic stresses trigger the disruption of transcription termination (DoTT), which results in a read-through transcript that extends to the next gene instead of stopping at the 3′-end of the previous gene [62]. The read-through transcripts are strongly enriched in chromatin and soluble nuclear extracts and therefore cannot be efficiently exported to the cytoplasm. Thus, DoTT substantially regulates gene expression by inhibiting RNA export, which is associated with an overall decrease in the level of protein [63]. Under salt stress, 10% of human-encoded proteins produce transcriptional read-through events, and the poly(A) signal intensity of these genes is usually below the average, which may be the underlying cause of the read-through [64]. HSV-1 infection was followed by a significant transcriptional read-through of the interferon regulatory factor gene *IRF1*, which is important for the immune response against viruses, suggesting that transcriptional read-through may be one of the methods by which the virus escapes from host immunity [63].

#### 2.2.3. Translation Efficiency

Most mRNAs perform their functions at the protein level, and the 3′UTR can also regulate protein expression by affecting translation efficiency. In addition to participating in the regulation of RNA stability, microRNAs, and RNA-binding proteins, long 3′UTR also inhibits protein translation efficiency [65]. In general, the 3′UTR of proliferating cells is shorter than that of differentiating cells in higher animals [66]; correspondingly, mRNA isoforms with shorter 3′UTR exhibit higher translation efficiency [67]. Yeast grown on rich medium tended to express shorter transcripts compared to that on a mini medium, and shorter 3′UTR correlated with up-regulation of genes participating in translation [33]. Under oxidative stress, C/EBPγ, the target of mTOR signaling, appears shortened in 3′UTR; short transcripts have high translational efficiency, and high levels of C/EBPγ expression control redox homeostasis [68]. Therefore, 3′UTR APA can also rapidly regulate the expression of genes by regulating translation efficiency in subtle changes in the cellular and surrounding environment.

**Figure 2:**
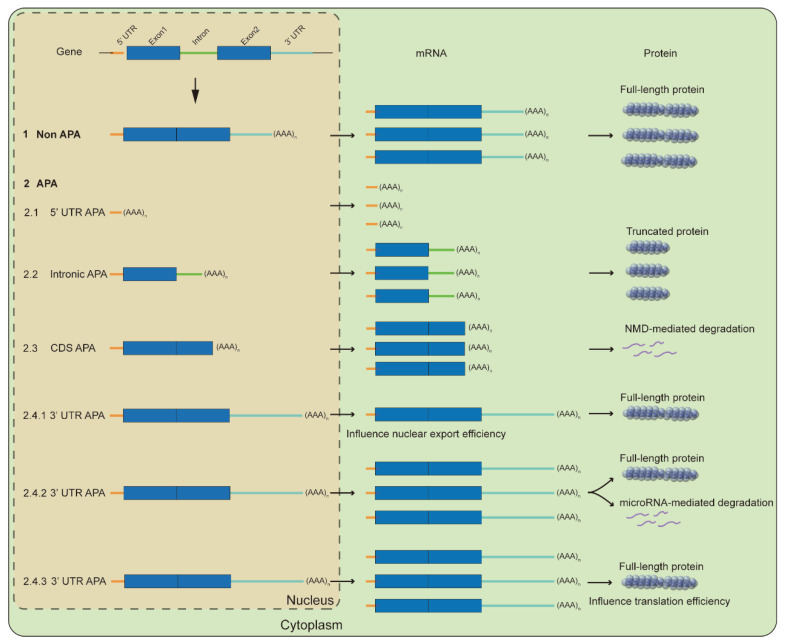
Molecular mechanisms for APA-mediated responses. Upstream-region APA often produces abnormally transcript-encoding short truncated proteins or is directly degraded by NMD-mediated pathway, thereby reducing the proportion of functional transcript. However, 3′UTR APA generally affects protein levels by influencing nuclear export, RNA stability, and translation efficiency.

In summary, the poly(A) site usage of many genes changes significantly under stress, such as the usage alteration of poly(A) site from the 3′UTR to the 5′UTR, introns and exons, and of course other positions in the 3′UTR. Genes usually escape from microRNA control due to the shortened 3′UTR and thus is up-regulated at the RNA level, which may facilitate the rapid response of resistance to stress, and 3′UTR shortening also improves translation efficiency and thus increases the expression at the protein level due to the presence of the binding motif in the 3′UTR for RNA-binding protein (Figure 2). The intronic APA and transcriptional read-through may down-regulate gene expression by reducing the ratio of functional transcripts or affecting the output of transcripts (Figure 2), and even transcriptional read-through may form long non-coding RNAs to regulate the expression of peripheral genes. At present, there are few studies on 5′UTR APA and exon APA, and they are also a strategy to reduce gene expression.

## 3. An Overview of the Role of APA in Response to Biotic and Abiotic Stresses

The regulation of gene expression is one of key steps for the development and response to environmental changes in eukaryotes. Plants require more precise regulation of gene expression than animals and yeast because they have smaller transcription units and intergenic regions [69]. Several recent reviews have summarized the role of APA on the regulation of plant growth and development [69,70,71]. Here, we focus on the role of APA in the plant stress response.

Stresses are commonly found in the environments where the organisms live, grow, and develop. Based on the characteristics of these stresses, they can be classified as biotic and abiotic stresses, which seriously affect the survival of plants and animals [72]. As sessile organisms, plants are more susceptible to stresses than animals. Throughout their life cycle, plants are constantly exposed to a variety of external stimuli. As a result, crop yields are affected to varying degrees by environmental stresses such as drought, salt, heavy metal, and hypoxic stresses. At the same time, plants also actively respond to environmental changes through the regulation of gene expression [73]. To overcome inevitable harsh environmental challenges, plants have evolved multiple gene-regulatory mechanisms to avoid injury. Here, we highlight the important role of APA in response to environmental changes, particularly in plants.

### 3.1. Hypoxic Stress

Oxygen is an inseparable component of life for all types of organisms, including plants, and flooding often affects oxygen availability for plants. A recent study showed the significant changes in poly(A) site selection for the transcriptome for *Arabidopsis thaliana* under hypoxic stress. Normally, 83% of the poly(A) sites are clustered at the 3′UTR, followed by at the CDS and introns for 11% and 5.6%, respectively, and cluster the least at the 5′UTR with 0.4%. However, a substantial up-regulation of the proportion of non-classical mRNAs was observed under hypoxic stress, with more than 10% of poly(A) sites located in the 5′UTR and introns being up-regulated at least two-fold and about 6% up-regulated at exons [73,74]. Transcripts that generate with poly(A) sites located in the CDS lead to the formation of abnormally long transcripts, thus affecting RNA stability and translation efficiency [75]. Transcripts that generate with intronic poly(A) sites also reduce RNA stability and translation efficiency and may encounter a stop codon in the intron and eventually express a truncated protein [76]. APA that occurs in the 5′UTR generally has no coding region and therefore cannot be translated into protein. They may function as long noncoding RNAs [77]. These non-classical mRNAs mentioned above reduce the proportion of full-length transcripts and may act as a negative regulatory strategy for gene expression, therefore regulating the balance of plant development and stress resistance.

### 3.2. Drought Stress

Water is one of the indispensable factors in plant growth, and drought severely hampers normal development and physiological metabolism in plants. A subset of 3′UTR extension events occurs in *Arabidopsis thaliana* under drought stress, and the proportion of 3′UTR extension events is significantly up-regulated with the increase in treatment time [78]. These 3′UTR extended transcripts represent less than 10% of the total transcriptome and are characterized by a weaker poly(A) signal in pre-mRNA than the others. These extension products do not appear to be functional as microRNA-binding sites, and the analysis of transcript length and expression level suggests that they may act as long noncoding RNAs to regulate the expression of their neighboring genes. About 43% of the extended 3′UTR transcripts overlaps with their downstream transcripts in the same or opposite transcriptional direction. When the 3′UTR extension is in the opposite direction to the downstream transcript, it generally acts as an antisense transcript to repress the expression level of their downstream genes, which could be explained by the mechanism of the RNA polymerase II (Pol II) collision model through the stalled Pol II transcriptional complex [79]. Conversely, when the 3′UTR extension is oriented in the same direction as the downstream transcript, it implies that the transcription reads into the promoter- or gene-coding region of the next gene, which creates opened and relaxed chromatin structures for neighboring genes, usually make them active [80]. The 3′UTR extension is also present in *fpa*, which is a loss-of-function mutant of a gene encoding a predicted RNA-binding protein FPA, and overlapped by 76% with drought treatment, implying that drought stress-induced 3′UTR extensions are highly correlated with the function of FPA for APA, which regulates flowering time by regulating *FLC* antisense APA. It is possible that more RNA-binding proteins are involved in the formation of the 3′-end in plants than in animals, as the proportion of highly conserved AAUAAA signals is greatly reduced in plants [81].

### 3.3. Salt Stress

Plants are affected by a number of abiotic stresses, including drought, salt stress, and heat stresses during their development. The main causes of salt injury in plants are ionic toxicity, osmotic stress, and oxidative damage [82]. A recent study found that the mutation in FIP1, which is a component of CPSF, exhibited less root length inhibition and less reduction in root meristem size under salt stress compared to the wild-type, showing an overall phenotype that is tolerant to salt stress [83]. Further studies revealed that the selection of poly(A) sites in the wild-type changed significantly under salt stress, as evidenced by a substantial up-regulation of the proportion parked on the 5′UTR and CDS, and the opposite on the 3′UTR. In contrast, the use of these non-canonical poly(A) sites did not change significantly in *fip1*. Mutations in FY and CPSF30, which are in the same CPSF complex as FIP1 does, similarly, alter the use of genome-wide poly(A) sites, including *AKR2* and *AT3G47610* [84]. The T-DNA mutants of *AKR2* and *AT3G47610* have an insertion site between the two poly(A) sites, which result in the use of only the proximal site, and both mutants showed higher seed germination rates than the wild-type under salt stress, again exhibiting the salt-tolerant phenotype. In addition, these two mutants also exhibit less sensitivity to ROS stress. The up-regulation of non-classical mRNA isoforms is also observed under various abiotic stresses in sorghum, which is at the expense of the down-regulation of the poly(A) site at the 3′UTR, with particularly drastic changes under the salt stress condition [85]. A comparison of APA genes and differentially expressed genes (DEGs) under different stresses revealed a partial overlap, with the highest overlap of 20% under salt stress, suggesting that APA may play a role in regulating the expression of some stress-responsive genes. *Eutrema salsugineum* showed stronger resistance to salt stress than *Arabidopsis thaliana*, and sequencing data showed that APA events occurred in some genes required for salt tolerance, such as *CIPK21* and *MAP3Kδ4*. *AtMAP3Kδ4* promotes the use of distal sites on the 3′UTR under salt stress, while *AtMAP3Kδ4* overexpression was also detected to enhance tolerance [86].

### 3.4. Nitrogen Starvation

Nitrogen (N) is one of the most important nutrients for plant growth, as it is one of the key components of proteins, nucleic acids, and phospholipids, which is mainly absorbed by the root system. In *Arabidopsis thaliana*, the wild-type plants showed shorter roots, while the root length was not changed obviously in *fip1-*2 under N starvation. At the same time, a large number of nitrogen-starvation response regulators, including *NRT2.4*, were rapidly induced. However, the expression levels of these regulators in *fip1* were significantly lower than that of wild-type plants, suggesting that *fip1* is insensitive to N starvation [87]. Further evidence indicates that the selection of the poly(A) sites in wild-type plants changes significantly under N starvation, mainly in the pattern of increased isoforms ending in the 5′UTR and CDS, and is decreased in introns, whereas the *fip1* restores 5′UTR APA and CDS APA, suggesting that FIP1 is required for the regulation of the polyadenylation of a large number of genes involved in nitrogen metabolism. For example, *NRT1.1*, an important nitrate transporter/sensor gene [88], undergoes 3′UTR APA in *fip1*, to promote the use of proximal sites. Furthermore, *cpsf3*0 exhibits similar defectivity in the nitrate response to *fip1*. CPSF30 has two proteins: CPSF30-L restores the defect of *cpsf30*, while CPSF30-S does not. Interestingly, both transcripts can be detected in wild-type plants, whereas the proximal sites located in introns are repressed in *fip1* or under N starvation. In addition, *CrNZF1*, which encodes a protein with three zinc finger motifs that are similar to CPSF30-L, is involved in nitrate signaling by regulating the length of 3′UTR of *NIT2* in *Chlamydomonas* [89]. These data indicate that both FIP1 and CPSF30 are important components of the nitrate-regulatory network in plants [28,90].

### 3.5. Temperature

Low temperature is also one of the important factors that reduces plant growth and crop yield. When plants are exposed to cold stress, C-repeat/DREB-binding factors (CBFs) are rapidly up-regulated in the short term, to activate the downstream cold-regulated genes (CORs), which protects plants against low temperature stress and improved freezing tolerance [91]. The three CBF genes, CBF1, CBF2, and CBF3, are distributed in tandem in the *Arabidopsis thaliana* chromosome, and the *cbf* triplet mutant exhibits slow growth and dwarfism phenotypes under normal conditions, while displaying a tall plant feature at low temperatures, implying that CBFs regulate the balance between plant growth and resistance to adversity stress [92,93]. In addition, the overexpression of CBFs can also adversely affect plant growth, and therefore, it is important to block the high level of CBF expression under low temperature stress [94]. Recent studies have revealed that the 3′UTR extension of *SVK* mRNA, a neighboring gene of *CBF1*, plays a key role in repressing the expression of *CBF1*. *SVK* and *CBF1* are transcribed in opposite directions, and the 3′UTR extension of *SVK* leads to the expression of antisense *CBF1* (*asCBF1*), which downregulates the expression of *CBF1*. The increased expression of *asCBF1* leads to the occupation of Pol II at the 3′-end of *CBF1*, which affects the process of transcription termination of *SVK*; thus, the 3′UTR extension of *SVK* maintains the upper limit of *CBF* expression and reduces the risk of adverse effects of CBF overexpression in plants [95].

### 3.6. Pathogens

It has been a challenge for viruses to turn off the expression of host genes and use the host expression system for themselves after invading host cells. One possibility is that the virus achieves its goal through extensive inhibition of mRNA splicing and RNA export [96,97], and now, the 3′-end-processing of mRNA appears to be a novel strategy for regulating host gene expression [98].

Plants lack the immune system to escape from unfavorable environmental conditions as animals do, and therefore face more varied types of complex invaders, in which case they have also evolved an effective mechanism to rapidly recognize pathogens and establish an effective resistance. Resistance genes (R genes), which are widespread in plants, play a key role in the immune response [99]. R genes are usually assembled in gene clusters, and genetic variation in basal resistance in plants is associated with polymorphisms of R genes [100]. *Japonica* rice showed higher disease resistance than *indica* rice, which was correlated with the APA of R genes between the two subspecies. Only about 20% of poly(A) sites of mRNA for R genes were located on the 3′UTR, which produces full-length transcripts in *indica* rice, while it is close to 70% in *japonica* rice [101]. In the case of the R gene *Xa1*, which specifically produces resistance to bacterial blight [102], the poly(A) site of *Xa1* in the 3′UTR is used to form functional full-length transcripts in *japonica* rice, whereas the poly(A) site in the CDS, whose transcripts may be rapidly degraded and fail to form functional proteins, is used in *indica* rice. In addition, *rTGA2.1*, which has a negative role in immunity, uses the proximal site on the 3′UTR in *indica* rice with an up-regulated expression level compared with that of *japonica* rice [103]. In contrast, the disease-resistance gene *RPM1* [104], which uses the proximal site on the 3′UTR, is substantially reduced in *indica* rice. The regulation of gene expression through 3′UTR APA is required for both *rTGA2.1* and *RPM1* in rice.

Recent studies have also identified a number of 3′-end-processing factors that is required for plant immune pathways, including CPSF30, FIP1, and PAPS1. CPR5 is a nucleoporin and works as a negative regulator in plant immunity; *cpr5* exhibits a dwarf and leaf-necrosis phenotype [105], and mutations in the 3′-end-processing factor *FIP1* suppress the phenotype of *cpr5*. FIP1 is a component of the CPSF complex, and mutations in another core component, such as *CPSF30*, similarly suppress the phenotype of *cpr5*, suggesting that the CPSF complex functions downstream of CPR5 to regulate plant immunity [106]. In addition, PAPS1 is a negative regulator of plant immunity, and the up-regulated immune response in *paps1* is suppressed by *eds1* [31]. *mips1* exhibited SA-dependent PCD, thus leading to leaf necrosis [107], and the mutation of *CPSF30* suppressed the phenotype of *mips1*, in addition to restoring the phenotype of other lesionmimic mutants including *lsd1*, *mpk4*, *cpr5*, and *cat2* [108]. *CBP60g*, a key regulator in the SA synthesis pathway, is a target gene of CPSF30, suggesting that plants regulate SA content by affecting RNA processing at the 3′-end of *CBP60g* pre-mRNA, thereby participating in the immune response [109].

### 3.7. ROS and ABA

Reactive oxygen species (ROSs) are by-products of cellular metabolism and usually act as signaling molecules to activate downstream reactions, while high levels of ROS induced by extreme environments increases the damage to cells [110]. Cadmium inhibits root growth through ROS signaling and cell-wall destruction, and APA switching events for genes required for ROS signaling and root development were also detected [111]. flg22 is a typical PAMP, which can trigger ROS bursts and pathogen accumulation after infecting plants [112]. Different *ERF4* mRNA isoforms are detected, including the predominant form, *ERF4-R*, a new long transcript *ERF4-IR*, and a new short transcript *ERF4-A* after flg22 treatment for *Arabidopsis thaliana* seedlings. ERF4-R is a transcriptional repressor with the positive regulation of ROS bursts, and interestingly, ERF4-A, produced due to the occurrence of APA, results in the lack of an EAR motif, which is converted into a transcriptional activator [113]. Therefore, an important function of ERF4-A produced under flg22 induction may be to eliminate ROS bursts under unfavorable environments and reduce the damage from ROS to plants. An oxidative stress-tolerant mutant, *oxt6*, was isolated and identified as a loss-of-function mutant of *CPSF30* in the model plant *Arabidopsis thaliana*. *oxt6* exhibited a dwarf phenotype under non-stress conditions, but showed stronger growth and longer roots than the wild-type under oxidative stress [114]. In addition, the rate of seed germination and the proportion of green cotyledons in *cpsf30* are significantly decreased when compared to the wild-type under ABA treatment, and the sensitivity to ABA could be reversed by complementation experiments; however, transgenic plants with mutations in the m6A (N^6^-methyladenosine)-binding YTH domain are not rescued in the phenotype, suggesting that the increased sensitivity of the *cpsf30* to ABA might be associated with m6A [115].

In summary, the aforementioned studies indicate that a wide range of APA events occur under biotic and abiotic stresses in plants, suggesting that APA may act as a positive post-transcriptional regulation in response to stress in plants (Table 1). However, more detailed molecular mechanisms remain to be explored.

## 4. Discussion and Future Perspectives

The rapid development of technologies for high-throughput sequencing in the last decade has given us the opportunity to deeply analyze the details of APA, but the exact mechanisms are still not well known, especially in plants, where further studies are to be carried out in this context. In this paper, we summarize the APA events that occur in plants and other eukaryotes in the face of adverse environments, which may be an act of self-help by the cell. In this process, APA factors, transcriptional complex components, and spliceosome components may influence the selection of poly(A) sites [116]. Of course, we believe that these components do not act individually and may be co-transcriptional, with the CTD domain of Pol II, which makes a prominent contribution due to its ability of linking transcription, splicing, and polyadenylation [117]. In addition, some RNA-binding proteins have been suggested to be associated with APA, such as HuR and FPA, with the former inhibiting the recruitment of CstF64 by binding GU-rich elements near the proximal site of pre-mRNAs, thereby promoting the use of its distal sites [57], and the latter affecting flowering time by regulating the use of proximal and distal poly(A) sites in *FLC* antisense transcripts [71]. These RNA-binding proteins mediate APA events, and we suggest that this feature may be more effective in plants because more than 80% of mRNAs in animals contains the most significant AAUAAA signal, whereas the percentage of this AAUAAA signal is notably reduced to about 10% for pre-mRNAs in plants, implying that although the overall molecular mechanism of APA may be similar among eukaryotes, the molecular mechanisms of polyadenylation in plants are more complicated in detail, as more regulatory factors may be involved [81].

For plants, it will also be a new concept in agricultural development to obtain favorable agricultural traits by regulating the selection of the APA site for genes that control these agricultural traits [118]. Rice *sdt* increased the yield by changing the number of panicle branches and tiller numbers, which was due to the up-regulation of the expression of OsmiR156h by the shortened polyadenylation tail [119]. In rice, *japonica* rice has a stronger disease resistance than *indica* rice, and it was found that there is a significant difference in the 3′UTR APA for some disease-resistance genes between them [101]. This also inspires us to use CRISPR Cas9 technology to edit the context of APA sites and use functional elements on the 3′UTR to rapidly up- and down-regulate gene expression, which is also meaningful for crop breeding.

## Figures and Tables

**Table 1:** Alternative polyadenylation mediates stress responses in plants.

Stress	Species	Target Genes	APA Types	Associated Polyadenylation Factors	References
Hypoxic	*Arabidopsis thaliana*		5′UTR APA CDS APA Intronic APA 3′UTR APA		[74]
Drought	*Arabidopsis thaliana*		3′UTR extension	FPA	[78]
Salt	*Arabidopsis thaliana* *Eutrema salsugineum* *Sorghum*	*AKR2* *AT3G47610* *CIPK21 MAP3Kδ4*	3′UTR APA	FIP1 CPSF30	[83,84,85,86]
N starvation	*Arabidopsis thaliana* *Chlamydomonas*	*NRT1.1* *CPSF30*	3′UTR APA Intronic APA	FIP1 CPSF30	[28,87,89,90]
Temperature	*Arabidopsis thaliana*	*SVK*	3′UTR extension		[95]
Pathogens	Rice *Arabidopsis thaliana*	*Xa1* *rTGA2.1* *CBP60g*	CDS APA 3′UTR APA	FIP1 CPSF30	[101,106,108]
ROS	*Arabidopsis thaliana*	*ERF4*	CDS *APA*	FPA CPSF30	[111,113,114]

## Data Availability

Not applicable.
